# Effects of Surface Modification on the Electrodialysis Performance of Anion-Exchange Membranes

**DOI:** 10.3390/membranes15120363

**Published:** 2025-11-30

**Authors:** Zhijuan Zhao, Qiang Dai, Shichun Feng, Jianhua Yang

**Affiliations:** Key Laboratory of Special Equipment Safety and Energy-Saving for State Market Regulation, China Special Equipment Inspection & Research Institute, Beijing 100029, China

**Keywords:** electrodialysis performance, anion-exchange membrane, surface modification

## Abstract

A commercial anion-exchange membrane was modified via the electrodeposition of different water-soluble polymers to study the effects of surface modification on electrodialysis performance. X-ray photoelectron spectroscopy and attenuated total reflectance Fourier transform infrared spectroscopy analyses showed that the different polymers were successfully electrodeposited on the membrane surface. The surface morphology and electrical resistance of the modified AEMs were almost unchanged. Contact angle and zeta potential measurements indicated differences in the surface hydrophilicity and surface charge density of the modified AEMs. The electrodialysis performance of the pristine AEM declined significantly in the presence of the foulant. In contrast, the electrodialysis performance of the AEMs modified with poly (vinylsulfonic acid, sodium salt) showed almost no decline and exhibited the best antifouling property in the presence of the foulant, followed by those modified with poly (sodium acrylate) and poly (vinyl alcohol). The results indicated that an increase in surface negative charge density and surface hydrophilicity increased the resistance of the modified AEMs to the foulant and improved their electrodialysis performance.

## 1. Introduction

The interest in desalination using electrodialysis is rapidly growing because of its many excellent properties, such as low energy consumption, high water recovery rates, and a high concentration ratio [1,2]. Recently, new applications of electrodialysis have been identified, such as nutrient recovery from wastewater sources [3,4,5,6,7]. However, the electrodialysis process still has technical limitations in spite of ongoing development, and further research is necessary to improve electrodialysis performance. Membrane fouling, particularly fouling of the anion-exchange membrane (AEM), is one of the most severe problems in the electrodialysis process [8,9,10,11,12,13]. For example, electrodialysis performance was observed to decline due to membrane fouling during the recovery of ammonia from swine manure [8]. Anionic polyacrylamide in industrial effluent could be deposited on the membrane surface and block the membrane’s free volume, hindering current transfer and reducing electrodialysis performance [13]. Almost all the organic foulants, such as humates, anionic surfactants, and proteins, in industrial wastewater have negative charge; hence, it is easy for these organic foulants to be adsorbed or deposited on the surface of the AEM by means of electrostatic and affinity interactions [14,15]. The membrane stack resistance exhibits an abrupt increase when membrane fouling occurs, causing energy consumption to increase and the desalination efficiency to decrease significantly. The membrane lifetime is shortened, and the electrodialysis process will eventually cease.

Membrane fouling is often thought to be affected by several factors: surface charge, surface hydrophilicity, and surface roughness [16]. Electrostatic interaction forces will promote membrane fouling if the foulants and the membrane have opposite charges. Surface hydrophilicity affects affinity interactions between the membrane surface and the foulant. Generally, a smoother surface is expected to have better antifouling properties because foulants are more likely to be adsorbed on a rougher surface. Moreover, Mitsuru et al. [17,18] examined the effects of differences in the chemical structure of AEMs on the membrane fouling phenomenon by preparing aliphatic and aromatic hydrocarbon-based AEMs; they indicated that adsorption of the foulant on the surface of the aliphatic AEMs was lower than that on the aromatic AEMs, which clarified the effects of affinity interactions on fouling behavior. Therefore, surface modification is an effective method to improve electrodialysis performance by improving the antifouling properties of commercial AEMs.

One proposed method for improving the antifouling properties of an AEM is to apply a coating of hydrophilic and negatively charged materials [19,20,21]. Grebenyuk et al. immersed an AEM into a solution of high-molecular-weight surfactant with three –OSO_3_^−^ groups to improve the antifouling performance of the modified membrane, using dodecylbenzene sulfonate (SDBS) and humic acid as the foulants [22]. Mulyati et al. modified an AEM by electrodepositing the anionic polyelectrolyte poly (sodium 4-styrene sulfonate) (PSS) on the membrane surface [20,21]. The polyelectrolyte layer on the membrane surface improved the antifouling properties of the modified membrane (with SDBS used as the foulant) by increasing the surface hydrophilicity and surface negative charge density. In previous work, the differences in the physicochemical properties of membranes modified with various polyelectrolytes were examined, and the surface properties of fouled membranes were also characterized [23], but the differences in the electrodialysis performance of the modified membranes were not examined.

In this study, three kinds of water-soluble polymers with different chemical structures were used to modify commercial AEMs to investigate the effects of surface modification on electrodialysis performance without and with a foulant. The physicochemical properties of the AEMs were characterized by means of XPS, ATR-FTIR, SEM, electrical resistance, contact angle, and zeta potential analyses. The effects of AEM surface modification on electrodialysis performance without and with a foulant were analyzed by determining the changes in conductivity of dilute solution in an electrodialysis experiment. The differences in the physicochemical properties and electrodialysis performance of the different AEMs were also analyzed.

## 2. Materials and Methods

### 2.1. Materials

**Figure 1:**
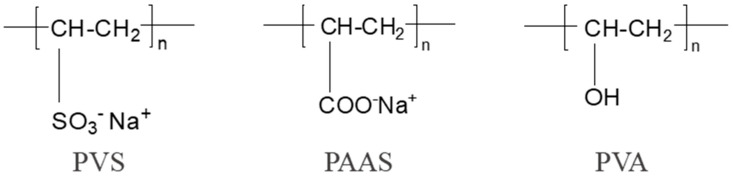
A schematic illustration of the chemical structures of the water-soluble polymers (PVS, PAAS, and PVA).

The AEMs and cation-exchange membranes (CEMs) used in the experiments were the Neosepta AMX and CMX (Astom Corp., Tokyo, Japan), and their characteristics are shown in Table 1. The water-soluble polymers were poly (vinylsulfonic acid, sodium salt) (PVS) (25 wt. % in H_2_O) (Sigma-Aldrich, Shanghai, China), poly (sodium acrylate) (PAAS) (average molecular weight, Mw = 5100) (Sigma-Aldrich), and poly (vinyl alcohol) (PVA) (average molecular weight, Mw = 89,000~98,000) (Sigma-Aldrich), and their chemical structures are shown in Figure 1. SDBS was chosen as a typical foulant. The other reagents (NaCl, Na_2_SO_4_, HCl, and Tris) were analytically pure. Ultra-pure water (18.2 MΩ·cm^−1^, Milli-Q, Millipore, Darmstadt, Germany) was used in all the experiments.

### 2.2. Surface Modification

**Figure 2:**
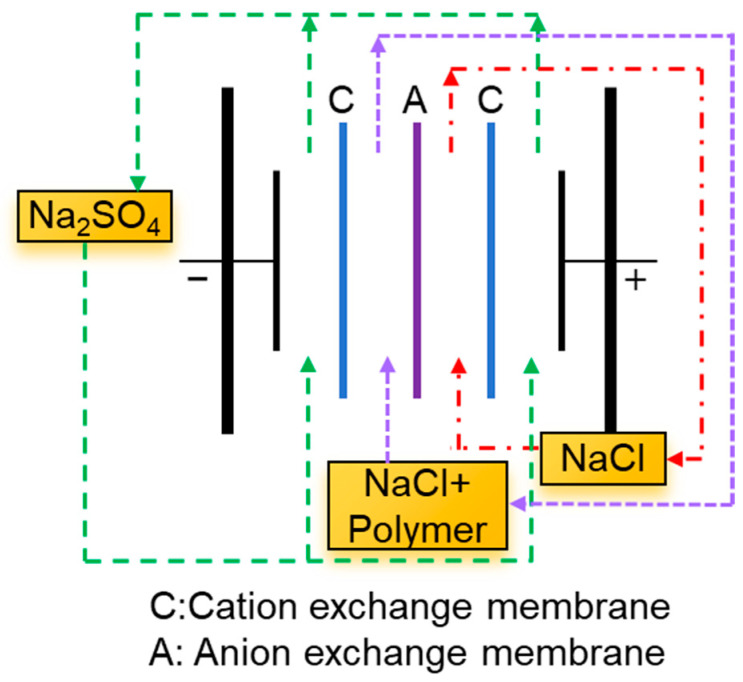
A schematic diagram of surface modification of the AEMs via electrodeposition.

The AEMs were modified via the electrodeposition of different polymers in an electrodialysis apparatus; a schematic diagram of the process is shown in Figure 2. Polymer (PVS or PAAS or PVA) solution with 0.1 mol/L NaCl as a supporting electrolyte in Tris-HCl buffer (pH = 7.0) was circulated through the dilute compartment, and 0.1 mol/L NaCl solution was circulated through the concentrate compartment, continuously driven by a peristaltic pump. The concentration of polymer was kept at 0.76, 0.55, or 0.26 g/L (for PVS, PAAS, and PVA, respectively) to ensure that the amounts of the functional groups were equal. A 0.1 mol/L Na_2_SO_4_ solution was pumped through the electrode compartment using a peristaltic pump. The surface modification process was performed at a current density of 5 mA/cm^2^ for 1 h. The surface of the AEM facing the dilute compartment was then modified. The membranes modified with PVS, PAAS, and PVA were named M1, M2, and M3, respectively, and the pristine AEM was labeled P0. After modification, the AEMs P0, M1, M2, and M3 were rinsed with ultra-pure water and then stored in ultra-pure water for further characterization.

### 2.3. Characterization of the AEMs

The surface chemical compositions of the AEMs were analyzed by means of X-ray photoelectron spectroscopy (XPS) using a 250Xi Escalab photoelectron spectrometer (Thermo Fisher Scientific, Waltham, MA, USA); Al Kα radiation (1486.6 eV) was used unless otherwise stated. Wide-scan spectra were obtained with a pass energy of 100 eV. Attenuated total reflectance Fourier transform infrared spectroscopy (ATR-FTIR) was used to characterize the surface functional groups of the pristine and modified membranes. The spectra were acquired on a T27-Hyperion-Vector22 spectrometer (Bruker, Bremen, Germany) in the range of 4000 to 600 cm^−1^ with 16 scans at a resolution of 4 cm^−1^ under ambient conditions. The surface morphologies of the AEMs were observed via field emission scanning electron microscopy (FE-SEM, Hitachi SU8020, Tokyo, Japan) at an accelerating voltage of 5 kV and a current of 10 μA. The electrical resistance of the AEMs was measured by using an LCR Meter (NF2321, Japan) set at 100 kHz and 100 mV in 0.5 mol/L NaCl at 25 °C. The contact angles of the AEMs were measured via the sessile drop method using a contact angle analyzer (DataPhysics, OCA20, Filderstadt, Germany). A 5 μL liquid droplet was deposited onto the membrane surface at a rate of 0.5 μL/s using a 1 mL syringe, and an image of the droplet was subsequently captured and analyzed. The value reported here is the average of five measurements. The zeta potential of the AEMs was measured using a zeta potential measurement analyzer (Anton Paar, Graz, Austria). Two identical membrane samples were sandwiched with a spacer in between and loaded into a clamping cell, forming a channel for electrolyte flow. The zeta potential was measured using a 1 mM KCl solution as the background electrolyte. The value was obtained as the average of four measurements.

### 2.4. Electrodialysis Experiments

Electrodialysis experiments in the absence and presence of the foulant SDBS were performed using an electrodialysis apparatus. The membrane stack contained two CEMs and one AEM (P0, M1, M2, or M3). The modified surface of the AEM (P0, M1, M2, or M3) was arranged to face the dilute compartment. The effective membrane area of each membrane was 55 cm^2^, and the intermembrane distance was 0.9 mm. The electrodialysis experiments were performed at a voltage of 4.00 V for 90 min. A volume of 500 mL of 0.1 mol/L Na_2_SO_4_ was used for the electrode solution. In the absence of SDBS, the initial solution in both the dilute and concentrate compartments was 500 mL of 0.1 mol/L NaCl, and the electrical conductivity of the solution in the dilute compartment was recorded by an on-line electrical conductivity meter. In the presence of SDBS, the initial solution in the dilute compartment contained 500 mL of 0.1 mol/L NaCl and 50 mg/L SDBS, and the initial solution in the concentrate compartment was 500 mL of 0.1 mol/L NaCl. The flow rate of the solution into the desalination chambers was 100 mL/min. The reduction rate difference for electrical conductivity in the dilute compartment without SDBS demonstrates the effects of surface modification on electrodialysis performance. The reduction rate differences for electrical conductivity in the dilute compartment with SDBS reflect the antifouling properties of the different AEMs. To further investigate the antifouling properties of the AEMs (P0, M1, M2, and M3), the AEMs were taken out of the apparatus after the electrodialysis experiments in the presence of SDBS, and their surface morphology was observed via FE-SEM.

## 3. Results and Discussion

### 3.1. Characterization of the AEMs via XPS, ATR-FTIR, SEM, and EDS

**Figure 3:**
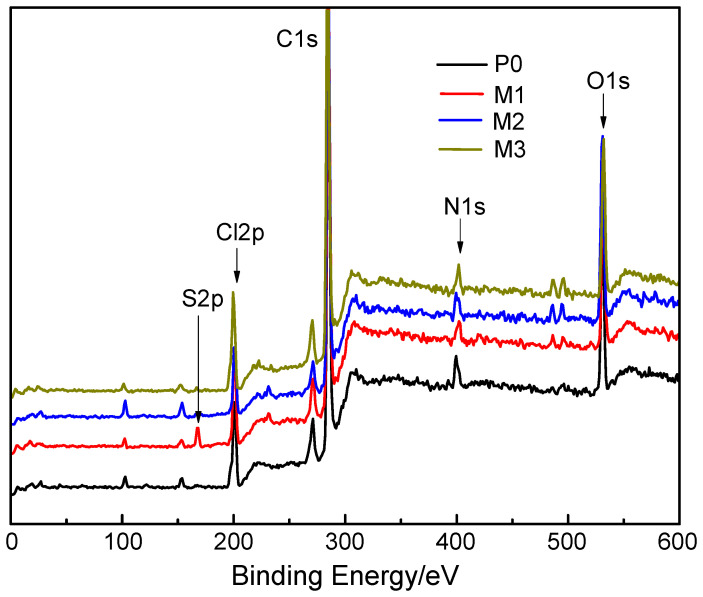
XPS spectra of the pristine and modified AEMs.

XPS survey scans of the AEMs were conducted to measure the chemical composition of the membrane surface, as shown in Figure 3. A new peak that was not observed for P0 appeared on the spectrum for M1. This new peak at 168.25 eV, corresponding to S2p, could be attributed to the –SO_3_H group contained in PVS. The existence of the S2p peak proved that PVS was electrodeposited on the membrane surface via electrostatic and affinity interactions. No new peaks appeared on the spectra for M2 and M3 because the elements contained in PAAS and PVA were the same as the partial elements of the base AEM. But the atomic concentration data presented in Table 2 show an evident change in the element contents of the different membrane surfaces. The change in the O/N atomic ratio could characterize the change in the surface chemical composition caused by the electrodeposition of PAAS and PVA because the pristine AEM contained quaternary ammonium groups as its active groups and the polymers used here did not contain nitrogen. The O/N atomic ratios of M2 and M3 were 4.03 and 4.11, respectively, both higher than that of P0, proving that PAAS and PVA were also electrodeposited on the membrane surface. These results indicated that the surface chemical composition of the membrane changed slightly after the electrodeposition of polymer on the membrane surface.

**Figure 4:**
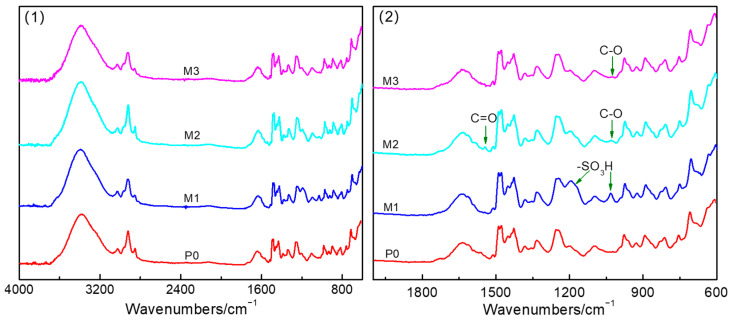
ATR-FTIR spectra in the ranges of 4000–600 cm^−1^ (**1**) and 2000–600 cm^−1^ (**2**) of the pristine and modified AEMs.

ATR-FTIR of the AEMs was performed to study the chemical structure of the membrane surface; the results are shown in Figure 4. Two new peaks at ~1190 cm^−1^ and ~1030 cm^−1^ appeared on the spectrum for M1, which were ascribed to the antisymmetric and symmetric stretching vibrational modes of the sulfonic group contained in PVS. Two new peaks at ~1560 cm^−1^ and ~1020 cm^−1^ appeared on the spectrum for M2, attributed to the stretching vibrations of C=O and C–O, respectively, which indicated that PAAS was adsorbed on the membrane surface. A new weak peak at ~1020 cm^−1^ appeared on the spectrum for M3, attributed to C–O in PVA, which indicated that PVA was also adsorbed on the membrane surface. These results were consistent with those obtained by means of XPS analysis.

**Figure 5:**
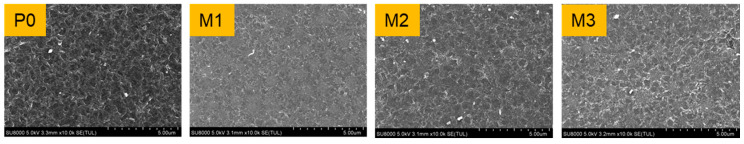
SEM images of the pristine and modified AEMs.

The surface morphology of the pristine and modified AEMs was observed via SEM; images with a magnification of 10,000 are shown in Figure 5. Slight morphological variations appeared on the surface of M1, and a very thin polyelectrolyte layer was electrodeposited on the membrane surface. For M2 and M3, almost no morphology changes were observed on the membrane surface. The results indicated that the electrodeposition was a mild modification method that did not significantly affect the surface morphology. This was because the amount of polymer adsorbed on the membrane surface was small, which was consistent with the results of the XPS analysis.

### 3.2. Membrane Electrical Resistance, Surface Hydrophilicity, and Charge Density

The electrical resistance of the pristine and modified AEMs was measured to investigate the effects of surface modification on the membranes’ electrochemical properties; the results are shown in Table 3. Compared with the pristine AEM, almost no changes occurred in the electrical resistance of any of the modified AEMs. It is well known that the molecular weights of the polymers are so large that they could not pass through the AEM. Under the force of an electric field, polymer accumulates on the surface of the AEM. According to the XPS and SEM analyses, the polymer layer electrodeposited on the surface of the AEM was thin and would not change the bulk properties of the modified AEMs. Therefore, surface modification with the polymers had almost no effect on the membranes’ electrical resistance.

**Figure 6:**
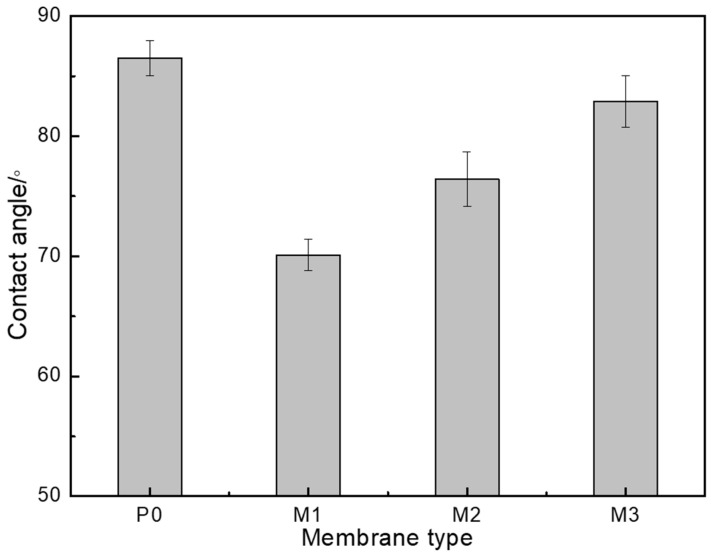
Water contact angles of the pristine and modified AEMs.

As shown in Figure 6, the water contact angle of P0 was 86.5 ± 1.46°, indicating the pristine AEM’s low affinity to water. When modified with the polymers, the AEMs tended to exhibit decreased contact angles to different extents. The contact angle of M1 decreased to 70.1 ± 1.31°, achieving the most significant improvement in surface hydrophilicity. The water contact angle of M2 was 76.4 ± 2.28°, showing a small improvement in surface hydrophilicity. However, the contact angle of M3 was almost unchanged with the electrodeposition of PVA.

Of the three polymers, PVS and PAAS belong to a special kind of polymers called polyelectrolytes. These two polyelectrolytes share some common characteristics with PVA in that their polyethylene chains are hydrophobic segments and their functional groups (–SO_3_H, –COOH, –OH) are hydrophilic groups. The polyanions formed by the polyelectrolytes when they are dissolved in solution could be attracted to the AEM through electrostatic interactions, but PVA is a nonionic polymer that only interacts with the AEM through weak affinity interactions. Hence, the amount of PVA adsorbed on the membrane surface was much smaller than that of the polyelectrolytes. Surface modification with PVA exhibited few effects on the hydrophilicity of the AEM. When the polyelectrolyte was adsorbed on the surface of the AEM through electrostatic interaction, the hydrophilic segments of the polyelectrolytes were exposed on the AEM surface. Because the electrostatic interaction force between the sulfonic acid group and the AEM’s quaternary ammonium group is much stronger than that between the carboxylic group and the quaternary ammonium group, the amount of PVS adsorbed on the membrane surface was much larger than that of PAAS. Besides this, the sulfonic acid group could exit by means of complete ionization, causing better surface hydrophilicity.

**Figure 7:**
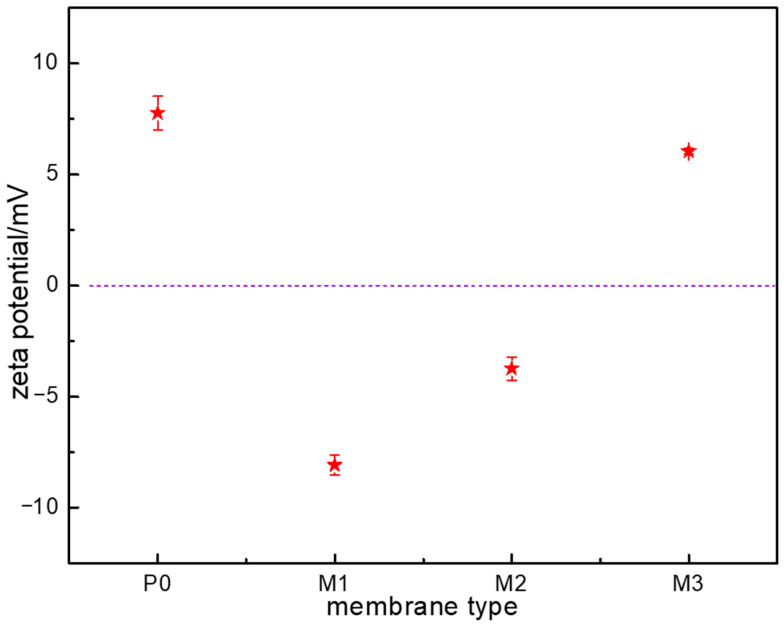
The zeta potential values of the pristine and modified AEMs.

The zeta potential values of the pristine and modified AEMs are shown in Figure 7. It is well known that the AEM contains quaternary ammonium groups as its active groups, and the zeta potential was measured at ~8.75 mV in 1 mmol/L KCl solution. When the AEM was modified with the polyelectrolytes, the surface charge density of M1 and M2 became negative because of the polyanions electrodeposited on the membrane surface. But the surface charge density of M3 was still positive after the electrodeposition of PVA. This was because the partial surface of M3 was covered with the nonionic polymer PVA, and the covered membrane surface showed neither the positive charge properties of the quaternary ammonium groups nor the negative properties. Hence, the zeta potential of M3 was still positive and lower than that of P0 because of the electrodeposited PVA. The zeta potential values of M1, M2, and M3 were ~−8.78 mV ~−3.74 mV, and ~6.74 mV, respectively. The absolute zeta potential value of M2 was smaller than that of M1, the reason for which might be that carboxylic acid was the weak electrolyte and the degree of ionization of PAAS was incomplete in solution. Even though PAAS polyanions were electrodeposited on the membrane surface, the measured zeta potential value was still low because of incomplete ionization of the carboxylic groups. However, the sulfonic acid group of PVS electrodeposited on the surface of the AEM was completely ionized in solution and exhibited a high negative charge density.

### 3.3. Electrodialysis Performance

**Figure 8:**
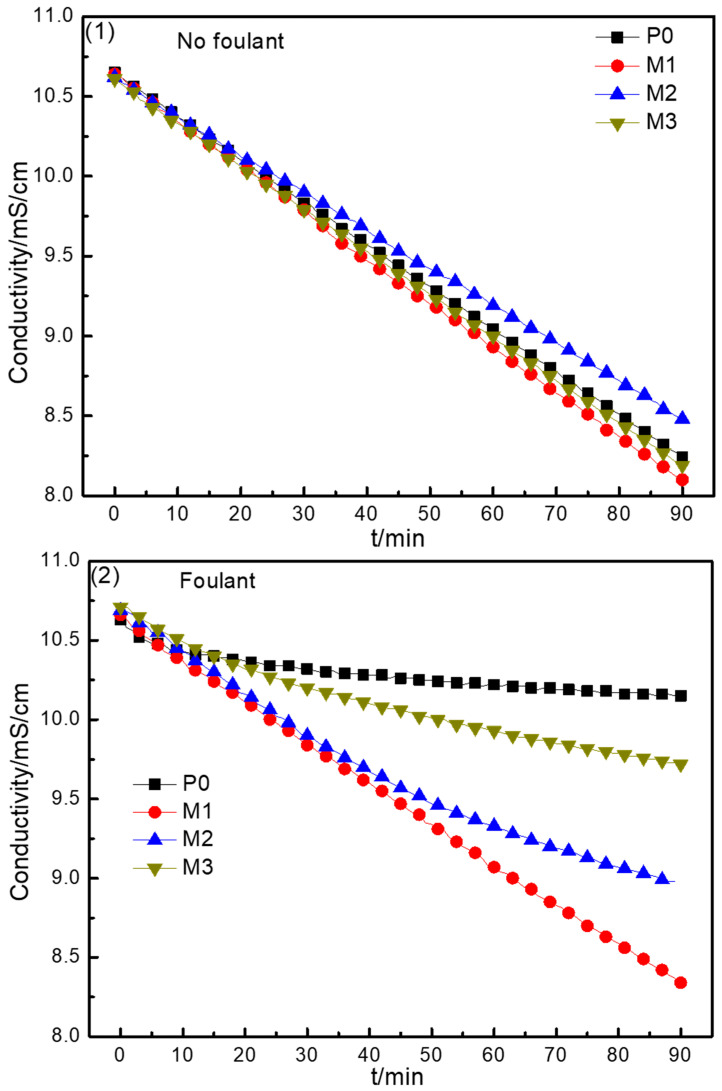
The electrical conductivity variations for the solution in the dilute compartment without foulant (**1**) and with foulant (**2**) in the electrodialysis process.

To examine the effects of surface modification on electrodialysis performance, desalination experiments in the absence of foulant were performed at a voltage of 4.0 V; the results are shown in Figure 8(1). There was almost no difference in the desalination rate between the modified AEMs and the pristine AEM in the same time period. The bulk properties of the modified AEMs could not be changed by polymer electrodeposition on the surface of the AEMs. The increase in the surface hydrophilicity and the change in the surface charge density of the modified AEMs would not affect the migration of ions either in solution or across the membrane. Hence, the ED performance was almost unaffectedby surface modification.

Figure 8(2) shows the electrodialysis performance of the pristine and modified AEMs when 50 mg/L of SDBS was included in the dilute compartment as a foulant. It is clear that the desalination rate decreased differently for the pristine and modified AEMs when SDBS was contained in the dilute compartment, as compared with the results shown in Figure 8(1). The reduction rate for electrical conductivity was very slow at the beginning of the experiment, with a value of approximately 10.1 mS/cm after 90 min using P0 with SDBS in the dilute compartment. This result indicated that it would be difficult to achieve transmembrane migration of ions because of severe membrane fouling, causing a decline in desalination properties. The desalination rate of M3 became slower after 10 min and that of M2 became slower after 50 min, as compared with the results shown in Figure 8(2). However, the desalination properties of M1 were almost unaffected throughout the whole electrodialysis process, and the final electrical conductivity value was only a little higher with SDBS in the dilute compartment than it was without SDBS, as shown in Figure 8(1). The results also reflected the antifouling performance of the AEMs: M1 showed the best antifouling properties, followed by M2, and the antifouling properties of M3 were the weakest. Comparing the two figures in Figure 8 clearly shows that surface modification of the AEMs improved their electrodialysis performance to different extents when the initial solution contained foulant.

**Figure 9:**
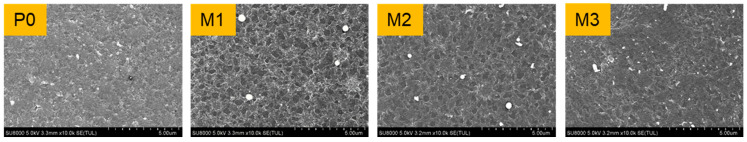
SEM images of the pristine and modified AEMs after electrodialysis experiments in the presence of the foulant SDBS.

Moreover, the surface morphology of the AEMs after the desalination experiments with the foulant SDBS was observed and is shown in Figure 9 to obtain a better perspective on the fouling resistance of the modified membranes. The surface morphology of the AEMs was not changed by surface modification, as shown in Figure 5. However, after the desalination experiments, the surface morphologies of the AEMs exhibited different changes to some extent. The surface of P0 was covered with a dense foulant layer, the surface of M3 was also covered but with a thinner foulant layer, and the surface of M2 was partially covered with a foulant layer. The surface morphology of M1 was similar to that before the desalination experiment, but particles were adhered to the membrane surface. This indicated that the antifouling properties of the modified AEMs improved to different extents, which improved their electrodialysis performance when the initial solution contained foulant. Consistent with the results of the desalination experiments, the antifouling property of M1 was the best, followed by M2 and M3. According to the surface hydrophilicity and surface charge of the modified membranes, an increase in membrane surface hydrophilicity and negative charge density leads to improved antifouling properties.

## 4. Conclusions

In this study, commercial AEMs were modified by electrodepositing polymers with different chemical structures on the membrane surface to examine the effects on the AEMs’ physicochemical properties and electrodialysis performance. The results confirmed that the different polymers were successfully electrodeposited on the membrane surface. The bulk properties of the modified AEMs, including their electrical resistance and surface morphology, exhibited almost no changes. However, the surface hydrophilicity and surface charge density of the three modified AEMs changed to different extents. M1 exhibited the best surface hydrophilicity, followed by M2 and M3. The surface charge density of M1 and M2 became negative, and the absolute value for M1 was higher than that for M2. Desalination experiments confirmed that surface modification had no effects on electrodialysis performance in the absence of foulant. However, surface modification did improve electrodialysis performance differently in the presence of the foulant SDBS because the surface modification layer on the AEM showed resistance to the foulant. The electrodialysis performance of M1 was the best, followed by that of M2 and M3. The results indicated that the chemical structure of the modification components, particularly the functional groups with charge, exhibited significant effects on the physicochemical and antifouling properties of the modified AEMs.

## Figures and Tables

**Table 1:** The characteristics of the membranes (Neosepta AMX and Neosepta CMX).

Membrane	Neosepta AMX	Neosepta CMX
Type	Strong base (Cl type)	Strong acid (Na type)
Burst strength/MPa	≥0.35	≥0.35
Thickness/mm	0.15	0.16
Temperature/°C	≤40	≤40
pH	0–14	0–14

**Table 2:** Surface atomic concentrations (%) of elements for the pristine and modified AEMs, obtained via XPS.

	Atomic %					Atomic Ratio
	Cl	C	N	O	S	O/N
P0	7.17	74.01	4.71	14.12	—	3.00
M1	6.14	76.44	2.92	12.61	1.88	4.32
M2	4.83	77.82	3.45	13.91	—	4.03
M3	7.62	78.53	2.71	11.13	—	4.11

**Table 3:** The electrical resistance of the pristine and modified AEMs.

Membrane Type	P0	M2	M3	M4
Electrical resistance/Ω·cm^2^	2.93 ± 0.037	2.97 ± 0.033	2.92 ± 0.017	2.89 ± 0.019

## Data Availability

The original contributions presented in this study are included in the article. Further inquiries can be directed to the corresponding author.
